# Orthrus: a Pumilio-family gene involved in fruiting body and dark stipe development in *Coprinopsis cinerea*

**DOI:** 10.3389/ffunb.2025.1633301

**Published:** 2025-07-30

**Authors:** Benedek Szathmári, Balázs Bálint, Botond Hegedüs, Máté Virágh, Zhihao Hou, Xiao-Bin Liu, Hongli Wu, Csenge Földi, Julien Gagneur, Johann Promeuschel, Árpád Csernetics, László G. Nagy

**Affiliations:** ^1^ Synthetic and Systems Biology Unit, Institute of Biochemistry, HUN-REN Biological Research Centre Szeged, Szeged, Hungary; ^2^ Doctoral School of Biology, Faculty of Science and Informatics, University of Szeged, Szeged, Hungary; ^3^ School of Computation, Information and Technology, Technical University of Munich, Garching, Germany; ^4^ Munich Center for Machine Learning, Munich, Germany; ^5^ Korea University, Seoul, Republic of Korea

**Keywords:** fruiting body formation, dark stipe, RNA-binding proteins, Pumilio, language model

## Abstract

Fruiting bodies of mushroom-forming fungi (Agaricomycetes) are complex multicellular structures whose formation is regulated by a developmental program that dynamically responds to environmental changes, such as light intensity. However, the genetic architecture and regulation of this developmental program are poorly known. Here, we characterize a novel Pumilio family gene, *ort2*, which influences fruiting body development, particularly the formation of dark stipes, a light-dependent alternative developmental trajectory. Phylogenetic analysis of this RNA-binding protein family in fungi revealed a distinct subfamily structure, with high conservation of each subfamily within Agaricomycetes. Reverse genetics experiments in the model species *Coprinopsis cinerea* revealed that *ort2* disruptants produced fruiting bodies, but were deficient in dark stipe formation, whereas the overexpression mutants produced significantly more dark stipes. The gene was named after Orthrus, the two-headed dog of classical mythology, based on rare but reproducible branching fruiting body phenotypes observed upon overexpression. Our findings reveal fruiting-related functions for *ort2*, a novel conserved RNA-binding protein, and may serve as a novel entry point for understanding the molecular basis of dark stipe development.

## Introduction

Fungal fruiting bodies are sexual reproductive structures that facilitate spore dispersal (Hibbett, 2007). They are complex multicellular (Knoll, 2011) – with a three-dimensional structure, in which not all the cells are in direct contact with the environment. They exhibit all key complex multicellular traits, such as intercellular communication, and a development that is orchestrated by a genetically encoded program (Kües, 2000; Nagy et al., 2018, 2023). They represent the most morphologically complex structures that fungi produce and have been a subject of intense research interest (Virágh et al., 2022).

Fruiting body development starts with the appearance of a dense hyphal aggregate, the primary hyphal knot, which appears in darkness, and turns into a secondary hyphal knot in the presence of light (Kües and Navarro-González, 2015). Hyphal knots represent the first three-dimensional developmental stage that fulfills all criteria of complex multicellularity (Knoll, 2011). Tissue differentiation primarily occurs during the primordium stages (P1–P5) and requires alternating light conditions (Kües, 2000; Kües and Navarro-González, 2015). While morphological aspects of the developmental process are well-described, their genetic underpinnings are poorly known, even though the development of fruiting bodies has been a central question in mycology.

Recent genomic and transcriptomic studies have significantly advanced our understanding of fruiting body morphogenesis in Agaricomycetes and complemented data from classical genetic studies (Kües, 2000; Ohm et al., 2011). Fruiting body development appears to utilize a deeply conserved yet flexible genetic toolkit (Krizsán et al., 2019; Nagy et al., 2023). Several genes, including those that encode transcription factors, cell wall remodeling enzymes, and signaling components, have been predicted to contribute to fruiting body development; however, the regulatory architecture remains incompletely resolved, since experimental characterization of most genes is lacking (Földi et al., 2024). Recent studies uncovered several key regulators of fruiting body development in model species such as *Coprinopsis cinerea* (Sakamoto et al., 2024; Masuda et al., 2016; Muraguchi et al., 2008; Muraguchi and Kamada, 1998) and *Schizophyllum commune* (Pelkmans et al., 2017; Beijen et al., 2025; Vonk et al., 2024), or commercially produced taxa, e.g., *Lentinula edodes* (Miyazaki et al., 2004) and *Agaricus bisporus* (Pelkmans et al., 2016). Most of these are transcription factors that bind DNA in a sequence-specific manner and regulate the expression of genes. However, the regulation of development likely also involves post-transcriptional and post-translational layers. The genes involved in these are currently unknown in mushroom-forming fungi.

In typical agaricomycete fruiting bodies, development proceeds from primordial stages to sporulation through stipe elongation and cap expansion. A characteristic, environment-dependent alteration of this developmental trajectory results in the formation of dark stipes or etiolated fruiting bodies (Tsusué, 1969). They are best studied in *C. cinerea*, which forms dark stipes in darkness; however, the phenomenon exists in several other species, often referred to by different names. Dark stipes are elongate structures that bear underdeveloped pileus and stipe tissues at their tip. They elongate via growth of the organ called nodulus (basal plectenchyma, basal shaft) (Kües, 2000; Chaisaena, 2009). Developmental stages up to P4 primordium can undergo etiolation when transferred to constant darkness (Subba, 2022). At the tip, the development of caps and stipes is arrested in the absence of light (Kües, 2000; Terashima et al., 2005). Upon light exposure, tissues at the tip can resume development and grow into a mature fruiting body (Tsusué, 1969); however, the original primordium is frequently aborted, and secondary primordia emerge from the dark stipe (Kües, 2000). In addition to darkness, elevated CO_2_ levels can also promote the formation of etiolated fruiting bodies (Subba, 2022).

High CO_2_ concentration and darkness trigger the formation of elongate fruiting bodies in other species, too. For example, *Flammulina velutipes, F. filiformis* (Yan et al., 2019), and *Pleurotus eryngii* (Zadražil, 1975) are cultivated under elevated CO_2_ conditions, to promote elongation and repress cap development. For these popular species, the elongated forms with smaller caps (called pinhead mushrooms in *Flammulina* species) are generally regarded to be of higher quality (Yan et al., 2019).

In earlier terminology, *C. cinerea* dark stipes are called pseudorhizae (Kües, 2000), since in nature, they appear as root-like structures within the substrate. The primary function of pseudorhizae is to push cap and stipe initials towards the light from below the surface of the substrate (*e.g.*, within dung) (Buller, 1958; Clémençon, 2012; Kües, 2000). In a broader perspective, the developmental program of dark stipes may represent an adaptation to escape enclosed, dark places, such as the interior of the substrate or cavities. The term pseudorhiza is widely used in mycology to describe similar, substrate-embedded, elongated forms in several species (Buller, 1958). Some of them (*e.g.*, *Hymenopellis radicata*, *Mycena galericulata*) are known to be functionally analogous (Buller, 1958) to *C. cinerea* pseudorhizae, but their developmental homology has not been proven.

Our current understanding of the molecular biology underlying dark stipe development is incomplete, limited to photoreception. The *dst1* gene is a WC1 photoreceptor, while *dst2* encodes a FAD/FMN-containing dehydrogenase (Subba, 2022). The inactivation of both *dst* genes results in a ‘blind’ phenotype, which refers to the formation of dark stipes even under standard (i.e., alternating light) fruiting conditions (Chaisaena, 2009).

Pumilio (Pum/PUF) constitutes a eukaryotic family of RNA-binding proteins with extensively studied members across animals, plants, and fungi alike. Pumilio proteins contribute to a broad spectrum of biological processes, such as embryonic patterning, stem cell maintenance, along with ribosome and mitochondrial biogenesis, among others (Nishanth and Simon, 2020). They play a critical role in post-transcriptional regulation, typically by destabilizing mRNA targets bound in a sequence-specific manner. mRNA destabilization is mediated via mechanisms such as inhibition of translation initiation, deadenylation, or microRNA activity (Nishanth and Simon, 2020). PUF RNA regulatory networks evolve dynamically; comparative analyses of predicted and experimentally validated targets across fungal species demonstrated recurrent rewiring (Wilinski et al., 2017).

In the fungal kingdom, the Pumilio proteins of *Saccharomyces cerevisiae* are the most extensively characterized. Yeast PUF proteins bind distinct subsets of transcripts, with defined recognition motifs (Gerber et al., 2004). Puf3p, one of the best-studied members of the family, localizes to the outer mitochondrial membrane and plays a role in mitochondrial biogenesis and motility (García-Rodríguez et al., 2007). The deletion of PUF3, as well as hyperphosphorylation of the encoded protein (Bhondeley and Liu, 2020) increases, whereas its overexpression decreases mitochondrial function, indicating that Puf3p is a negative regulator. Additionally, Puf3p was also reported to contribute to adaptation to metabolic changes via switching the balance in translational flux between mitochondrial and cytosolic ribosome biogenesis (Wang et al., 2019). The ortholog in *Schizosaccharomyces pombe*, Puf3, was shown to function via the Ccr4-Not complex (Webster et al., 2019). An analysis comprising 42 fungal (four basidiomycete) species indicates the mitochondrion-related function is an apomorphy of the Saccharomycotina clade (Jiang et al., 2010). The study of Wilinski et al. (2017) revealed that the *Neurospora crassa* Puf3p ortholog binds a similar motif to that of *S. cerevisiae* Puf3, but with a cytosine in a central position in half of the target sequences, instead of an adenine. In *Cryptococcus neoformans*, the Puf3 ortholog Pum1 serves as a pleiotropic regulator of sexual development, including filamentation and basidium maturation (Liu et al., 2018). In this species, the maintenance of the vegetative phase requires the autorepression of *PUM1* (Kaur and Panepinto, 2016), with a longer, yeast phase-specific transcript bearing the recognition motif of its own product. Pumilio proteins have been reported to have dynamic expression patterns during fruiting body formation of complex multicellular Agaricomycetes (Krizsán et al., 2019; Merényi et al., 2020; Nagy et al., 2023); however, whether these proteins contribute to complex development has not been experimentally studied until now.

In this study, we analyze Pumilio protein-encoding genes in *C. cinerea* and select one member, referred to as *ort2*, that is orthologous to *S. cerevisiae PUF3* and *C. neoformans PUM1* for functional investigation. We show that Pumilio proteins form distinct and conserved subfamilies in the Agaricomycetes, and that *ort2* influences fruiting body development in this species. We detected the largest impact of *ort2* on dark stipe development and attempted to decipher the mechanistic bases by which it influences development.

## Materials and methods

### Strain, buffers and culture conditions

We used the self-compatible, *para*-aminobenzoic acid auxotrophic AmutBmut1 *pab1-1* #326 strain of *C. cinerea* for genetic modification and as a control in all experiments. The fungus was maintained on solid YMG (yeast-malt-glucose) medium. All media and buffers used for culturing and transformation (YMG, minimal medium, regeneration medium, top agar, PEG/CaCl_2_ solution, MM and MMC buffers) were prepared as described by Dörnte and Kües (2012). Oidiation was induced by culturing at 37°C under constant white light. After transformation, we selected for the complementation of PABA auxotrophy using synthetic minimal medium.

### Phylogenetic analyses

Homologs of *ort2* were retrieved by blastp (e < 10^-5^) and an InterPro search (search terms: IPR001313, IPR033133) from a proteome dataset comprising 13 agaricomycete species, two additional basidiomycetes (*Cryptococcus neoformans*, *Ustilago maydis*) (Nagy et al., 2023) complemented by two ascomycete yeasts, *Saccharomyces cerevisiae* and *Schizosaccharomyces pombe*. The proteome of *Phanerochaete chrysosporium* is included in two versions. For our model species, *C. cinerea* version 2 was used. We refer to *C. cinerea* proteins based on the annotation of Hegedüs et al. (2025) (https://mushroomdb.brc.hu/); the IDs we supply can be found in the JGI annotation as gene (model) names (https://mycocosm.jgi.doe.gov/Copcin2/Copcin2.home.html). Homologs fitting the HMM of IPR040000 were excluded, as these are orthologs of the nucleolar protein Nop9 of *S. cerevisiae* (*C. cinerea* protein: 438228). Retained homologs were aligned by PRANK v.170427 (Löytynoja, 2014) by using the -F parameter and a maximum likelihood guide tree inferred in preliminary analyses by RAxML version 8.2.12 (Stamatakis, 2014), from a multiple sequence alignment generated by Mafft-linsi v7.453 (Katoh and Standley, 2013). The PRANK alignment was used for tree inference and bootstrapping by RAxML version 8.2.12, under the WAG model with gamma-distributed rate heterogeneity. Branch support was assessed by 500 non-parametric rapid bootstrap replicates. Trees were visualized in FigTree v1.4.4 (http://tree.bio.ed.ac.uk/software/figtree).

### Design of molecular tools

We intended to perform gene deletion through Cas9 cleavage followed by homologous repair, and overexpression via ectopic integration. We designed two crRNAs and a plasmid (p.Δ*ort2*) as a repair template, and another plasmid (p.*ort2*OE) was constructed to generate overexpressing strains (**Supplementary Figure S2**). The plasmids, primers and crRNAs were designed using SnapGene version 4.3.11 (https://www.snapgene.com/). The sequences of primers and crRNAs are listed in **Supplementary Table S1**. Both plasmids possess a backbone derived from pUC19 and carry the positive selection marker gene *pab1-1* with its promoter and terminator regions (CcPAB1) amplified from the pMA412 vector (Stanley et al., 2014). p.Δ*ort2* carries the marker gene between homologous arms (1 kb sequences upstream and downstream of the target sequence). p.*ort2*OE carries the *ort2* gene driven by the promoter of a heat shock protein gene, *Cc.hsp3* (ID: 423239), given that the promoter of its ortholog has been shown to be useful for overexpression in *Schizophyllum commune* (Ohm et al., 2011).

### Plasmid assembly and preparation

Fragments were amplified by Phusion Plus DNA polymerase (Thermo Fisher Scientific) using overlapping Gibson primers. Plasmids were assembled using the Gibson Assembly Cloning Kit (New England Biolabs, USA), following the manufacturer’s instructions. The ligation mixture was introduced into competent NEB5α cells following the protocol of Rabe and Cepko (2020), and the plasmids were purified from the cultures of colonies for which the presence and proper composition of the plasmids had been confirmed, using the NucleoBond Xtra Midi kit (Macherey-Nagel, Germany).

### Protoplasting oidia

Oidia were harvested from 5-day-old cultures grown under constant light, by washing with distilled water, filtering through a 40 µm cell strainer, and pelleting by centrifugation (1000 g, 10 min). Pellets were washed with 5 ml MM buffer, pelleted again, and resuspended in 1 ml sterile-filtered MM buffer containing ~20% w/v VinoTaste Pro (Novozymes, Denmark). Protoplasting was carried out by shaking oidia at 70–90 rpm in an incubator at 36°C for 3–4 hours. Once the majority of oidia turned into spherical protoplasts, the process was terminated by adding 5 ml MMC buffer, and the protoplasts were collected (1000 g, 15 min).

### PEG-mediated transformation of *C. cinerea* protoplasts

The pelleted protoplasts were resuspended in 100 µl MMC buffer. We added 10 µg plasmid DNA; in the case of the knockout transformation, the two RNP complexes [assembled as described by Pareek et al. (2022)]; and for both transformations, 30 µl PEG-CaCl_2_ solution. The cells were incubated on ice for 30 min, then 0.5 ml PEG-CaCl_2_ solution was added, and the mixture was incubated at room temperature for 10–20 minutes. After adding 20 ml MMC buffer, the solution was gently mixed, and the cells were pelleted (1000 g, 15 minutes). The cells were resuspended in 1 ml MMC buffer, mixed with top agar, and poured onto regeneration medium. Colonies were collected onto selective minimal medium plates. Transformants were checked by PCR, amplicon sequencing, and RT-qPCR. To obtain DNA template for the PCR reactions, vegetative mycelium was lysed thermally as described by Dörnte and Kües (2012). Amplicon sequencing was conducted commercially (Microsynth, Switzerland) after purification using the NucleoSpin Gel and PCR Clean-up kit (Macherey-Nagel, Germany). To purify mixed-genotype cultures, we prepared single-spore cultures from oidia.

### RNA purification and RT-qPCR

Cultures grown on cellophane were homogenized in a mortar with liquid nitrogen. RNA extraction was performed using the Quick-RNA MiniPrep kit (Zymo Research, USA) following the manufacturer’s instructions. We screened the overexpressing mutant candidates by RT-qPCR. The colonies of the transformants and the wild-type controls were exposed to 42°C heat shock for 2 hrs before sample collection. Reverse transcription was carried out with the RevertAid First Strand cDNA Synthesis Kit (Thermo Fisher Scientific), and qPCR reactions were performed using SsoAdvanced Universal SYBR Green Supermix (BioRad), following the manufacturers’ instructions. RNA extracted from the wild-type strain was used as a control, and the β-tubulin gene (protein ID: 393528) was chosen as the reference gene. Expression changes were calculated using the formula FC = 2^−ΔΔCt^ (Livak and Schmittgen, 2001). qPCR was conducted in six technical replicates: two reactions per experiment, repeated three times starting from the same RNA extracts.

### Phenotyping of fruiting bodies

Since light- and dark-grown colonies of *C. cinerea* produce fruiting bodies of different morphology, we investigated fruiting under both conditions. Strains were inoculated onto ‘half-sugar’ YMG (containing 2 g/L glucose) (Muraguchi et al., 2015), incubated in darkness at 28°C for six days, then half of the plates were transferred to alternating light conditions (12 hrs light, 12 hrs dark; Panasonic FL40SS-ENW/37 light bulb), while the rest were kept in darkness. Both groups were further incubated at 28°C for two weeks. After several smaller-scale phenotyping experiments, fifty plates of the deletion mutants and one hundred of the selected overexpression strains were phenotyped and compared to a wild-type control group. For the overexpression mutants, after the additional two-week incubation, we quantified fruiting, along with making morphological observations. We counted etiolated fruiting bodies (dark stipes) longer than 1 cm per Petri dish formed by dark-grown colonies, and structures that had at least started to elongate (P4 and older) in the case of plates exposed to alternating light. Phenotyping experiments were also conducted in jars containing 150 ml ‘half-sugar’ YMG with 6 g/L agar, with the same protocol.

### Assessment of relative mtDNA abundance

Following the method described by Venegas et al. (2011) and Galeota-Sprung et al. (2022), we performed qPCR on a nuclear (β-tubulin gene, JGI protein ID: 393528) and a mitochondrially encoded gene (NADH dehydrogenase subunit 1, protein ID: 384263), using DNA templates extracted from an *ort2*OE, four Δ*ort2* mutants, and the wild-type strain, by using the Blood & Cell Culture DNA Maxi Kit (Qiagen, Germany). We used five extracts from all the strains; and for each sample, two technical replicate qPCR reactions were performed. qPCR was carried out in the same way as described above.

### Functional studies

Research Group Julien Gagneur (Munich Centre for Machine Learning) provided us with a list of putative Ort2 recognition motifs and mRNA targets, predicted by their species-aware DNA language model (Karollus et al., 2024). We utilized the hits belonging to the most significant motif. The list contains 772 Okayama 7 genes; 655 remain when converted to AmutBmut identifiers. A Gene Ontology (GO) term enrichment analysis was performed on the AmutBmut gene set. A FIMO (Grant et al., 2011) search was also conducted for UTRs of *C. cinerea* (Hegedüs et al., 2025), using the *S. cerevisiae* Puf3p recognition motif PWM as described by Gerber et al. (2004). We aimed to select the top 100 most significant hits; however, due to the identical *p*-values among several entries, a total of 116 were included. We also generated three mitochondrial gene sets. Okayama 7 genes with a mitochondrial GO annotation were downloaded from QuickGO (https://www.ebi.ac.uk/QuickGO) and converted to AmutBmut identifiers. Mitochondrial gene sets were also obtained based on predicted subcellular localization. We employed TargetP 2.0 (Almagro Armenteros et al., 2019) and DeepLoc 2.0 (Thumuluri et al., 2022) to identify proteins potentially localizing to mitochondria. Hits with 0.7 or higher probability scores were considered significant. An overlap analysis was conducted on the five gene sets. An Euler diagram was drawn using the eulerr R package. For predicting the subcellular localization of Ort2, we used DeepLoc 2.0 (Thumuluri et al., 2022) as implemented at https://services.healthtech.dtu.dk/ and WolfPSort (Horton et al., 2007) at https://wolfpsort.hgc.jp. Both software were run with default parameters and the setting ‘slow’ for DeepLoc 2.0 and ‘fungi’ for WolfPsort.

## Results

### Pumilio proteins form five conserved subfamilies

Through BLASTp and InterPro searches, we collected the Pumilio protein sequences from the proteomes of 13 agaricomycete species, two additional basidiomycete species (*Cryptococcus neoformans*, *Ustilago maydis*) (Nagy et al., 2023), and two ascomycete yeasts, *Saccharomyces cerevisiae* and *Schizosaccharomyces pombe*. We identified 4 to 7 Pumilio proteins in the examined species of Basidiomycota, which is similar to copy numbers reported for Ascomycota (Matsushima et al., 2007). In *Coprinopsis cinerea*, six proteins that possess Pumilio-family RNA-binding repeat domains (IPR001313) were found. *C. cinerea* 438228 was excluded from the analyses, since its sequence is highly divergent, contains another domain (IPR040000), and, as an ortholog of the nucleolar Nop9 of *Saccharomyces cerevisiae*, it might function differently. Nop9 orthologs also formed a distant subfamily in previous studies (Najdrová et al., 2020).

**Figure 1:**
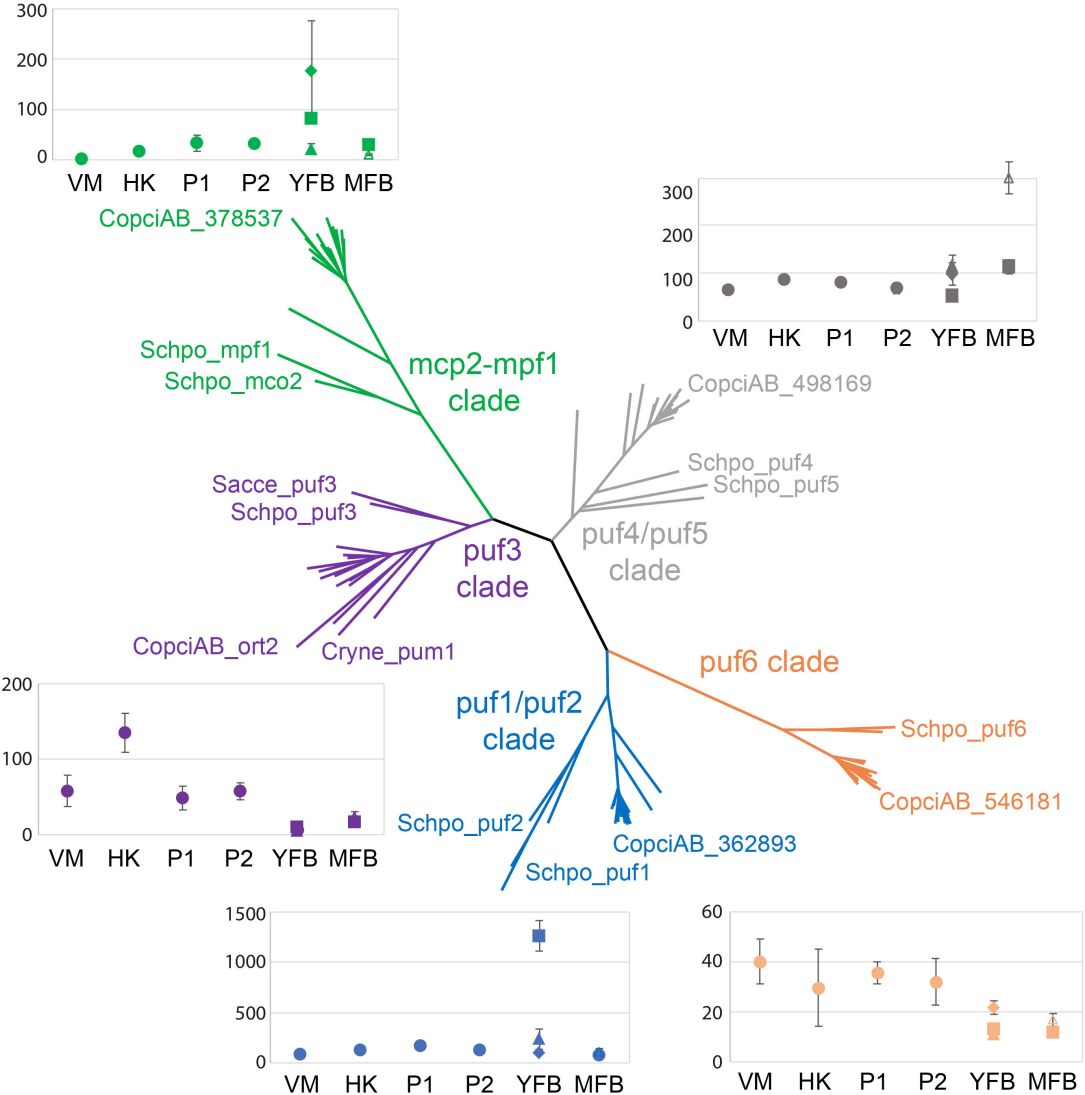
Unrooted maximum likelihood gene tree of fungal Pumilio proteins and expression dynamics of *Coprinopsis cinerea* Pumilio genes. The phylogenetic analysis comprises protein sequences from 17 fungal species. Pumilio proteins form five conserved subfamilies. Subfamilies are named after *Sch. pombe* proteins. Clade colors match those of the expression diagrams of the corresponding *C. cinerea* genes. In each clade, only terminal nodes belonging to *C. cinerea*, *Cr. neoformans*, *S. cerevisiae*, and *Sch. pombe* are labeled by JGI identifiers or common names. For more details, see **Supplementary Figure S1**. For the expression plots, we used Krizsán et al.’s RNA-Seq dataset (2019). The developmental stages are as follows: VM, vegetative mycelium; HK, hyphal knot; P1, stage 1 primordium; P2, stage 2 primordium; YFB, young fruiting body; MFB, mature fruiting body. In earlier stages (VM to P2), bulk expression values are shown (indicated by filled circles), in more developed stages (YFB and MFB), tissue-specific expression is plotted. In the YFB stage, squares, triangles, and diamonds indicate expression in stipe, cap, and gills, respectively. In the MFB stage, squares similarly indicate expression in the stipe tissue, while empty triangles mark combined cap and gill expression. Expression values are expressed as CPM on all plots.

The Pumilio family formed 5 clearly discernible and strongly supported (maximum likelihood bootstrap > 70%) subfamilies, each of which contained *S. cerevisiae* and *Sch. pombe* sequences, indicating conservation of major Pumilio subfamilies retained across Asco- and Basidiomycota (**Figure 1**; **Supplementary Figure S1**). Each subfamily harbors a single protein of each agaricomycete species, with few exceptions (e.g., two *Pleurotus ostreatus* paralogs in clade puf4-puf5). Subfamily topologies mostly reflect the species tree (Naranjo‐Ortiz and Gabaldón, 2019): agaricomycete proteins group together, while more basal branches represent the two ascomycetes and the two basidiomycete species outside Agaricomycetes. This indicates highly conserved evolutionary trajectories and low overall divergence within subfamilies. Subfamilies differ from each other considerably, as indicated by the long branches separating them. *C. cinerea* 354109 (CopciAB_ort2) grouped with Puf3 of *Sch. pombe*, Puf3p of *S. cerevisiae*, and Pum1 of *Cryptococcus neoformans* (**Figure 1**), whose function has been investigated (Webster et al., 2019; Liu et al., 2018), especially that of *S. cerevisiae* Puf3p (García-Rodríguez et al., 2007).

We inspected the expression dynamics of the five *C. cinerea* genes encoding Pumilio proteins in Krizsán et al.’s RNA-Seq dataset (2019) (**Figure 1**). The expression of *C. cinerea* 354109 (CopciAB_ort2) peaks in the hyphal knot (HK), the earliest stage of fruiting body formation (Kües and Navarro-González, 2015), suggesting it has functions in the early developmental events. We note that Xie et al.’s data (2020) indicate a maximum expression in vegetative mycelium; however, both datasets agree that its expression decreases as the developmental program progresses. For the phylogenetic and expression-related reasons discussed above, we selected *C. cinerea* 354109, later named *ort2*, for experimental characterization.

### Disruption of *ort2* affects fruiting body development

Using the RNP-mediated CRISPR-Cas9 genome editing technique (Pareek et al., 2022), we generated four Δ*ort2* disruptant strains (see the plasmid in **Supplementary Figure S2a**). The lack of *ort2* expression was demonstrated by RT-qPCR (**Supplementary Table S2**). Though we used two crRNAs, instead of complete deletion, we could achieve only gene disruption. The disruptant genomes were not screened for off-target integrations, given that the phenotypic similarity of the independent disruptant strains implies the observed characters likely result from the intended disruption of *ort2* rather than off-target effects.

**Figure 2:**
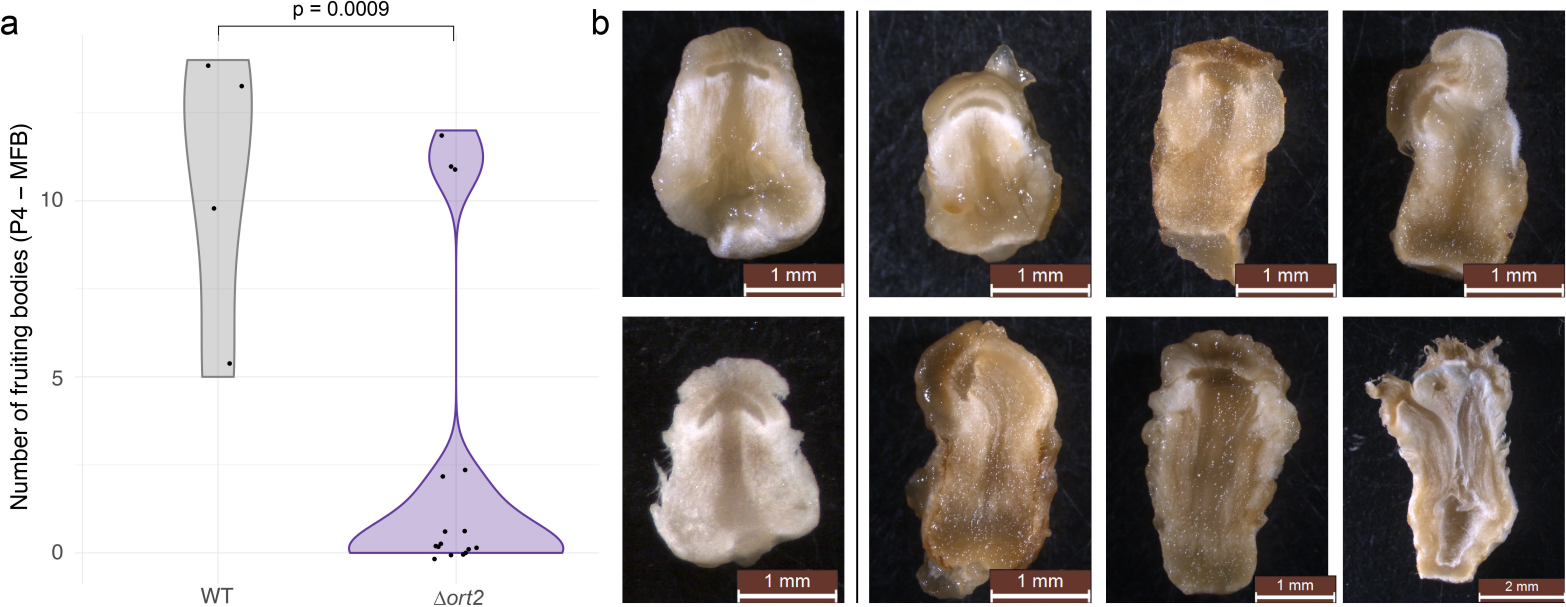
Phenotypic characterization of Δ*ort2* disruptants. **(a)** Violin plot: total number of fruiting bodies in stages from P4 to MFB on light-grown jar cultures of the wild-type and Δ*ort2* strains. **(b)** Cross-sections of wild-type (left panel) and Δ*ort2* (right panel) primordia.

Phenotyping of the four Δ*ort2* disruptants revealed no differences in vegetative growth patterns and oidiation relative to the parent strain (not shown), whereas fruiting body and dark stipe development seem to be impaired to a degree (**Figure 2**), the latter process considerably. Grown under alternating light conditions in jars, stage 1 primordia appeared at least 3 days later on disruptant cultures than on the wild-type ones, and no Δ*ort2* fruiting bodies reached maturity (i.e., failed to produce basidiospores and deliquesce). We counted structures whose stipes had already started to elongate, including P4 and P5 stage primordia (Kües and Navarro-González, 2015), as well as young and mature fruiting bodies, together on four wild-type cultures and 16 Δ*ort2* jar cultures (**Figure 2a**). On average, on wild-type cultures 10.5; while on Δ*ort2* cultures 1.875 such structures formed, which is a significant difference (*p* = 0.0009, two-sample t-test); although we acknowledge the limited power of the analysis due to the low number of data points and the presence of outliers. We conducted a larger-scale experiment with 20 wild-type and 20 mutant jar cultures. The wild-type cultures formed 16.05 ± 9.56 fruiting bodies (P4 to MFB), while none of the disruptants produced any fruiting bodies or initials, indicating the decreased readiness of the mutants for fruiting compared to the parent strain. No consistent difference was observed in the tissue structure of primordia; however, many of them exhibited diverse abnormalities (**Figure 2b**). When cultured under constant darkness on plates, wild-type cultures produced dark stipes, whereas the Δ*ort2* cultures did not. Across four wild-type jar cultures, a total of 34 dark stipes were counted, approximately 20 of which exceeded 1 cm in length/height. In contrast, on 16 Δ*ort2* jar cultures, only three dark stipe-like structures were observed altogether (all measuring less than 1 cm), along with some rudimentary structures that failed to elongate. This indicates that Δ*ort2* strains are capable of producing dark stipes, though to a considerably lower extent.

### Overexpression of *ort2*

Utilizing an ectopically integrating plasmid (**Supplementary Figure S2b**), additional copies of *ort2* were introduced into the genome of the AmutBmut strain, under the control of the *hsp3* heat shock promoter. RT-qPCR was performed to measure *ort2* expression in mycelia grown at 28°C (**Supplementary Table S3**). We selected the strain with the highest expression change (*ort2*OE(10); FC = 4.14 ± 2.79), along with another overexpression strain (*ort2*OE(7); FC = 1.28 ± 0.65) for preliminary phenotyping. After confirming the phenotypes were consistent across the independent mutants in preliminary experiments, *ort2*OE(10) was characterized further in larger-scale experiments.

**Figure 3:**
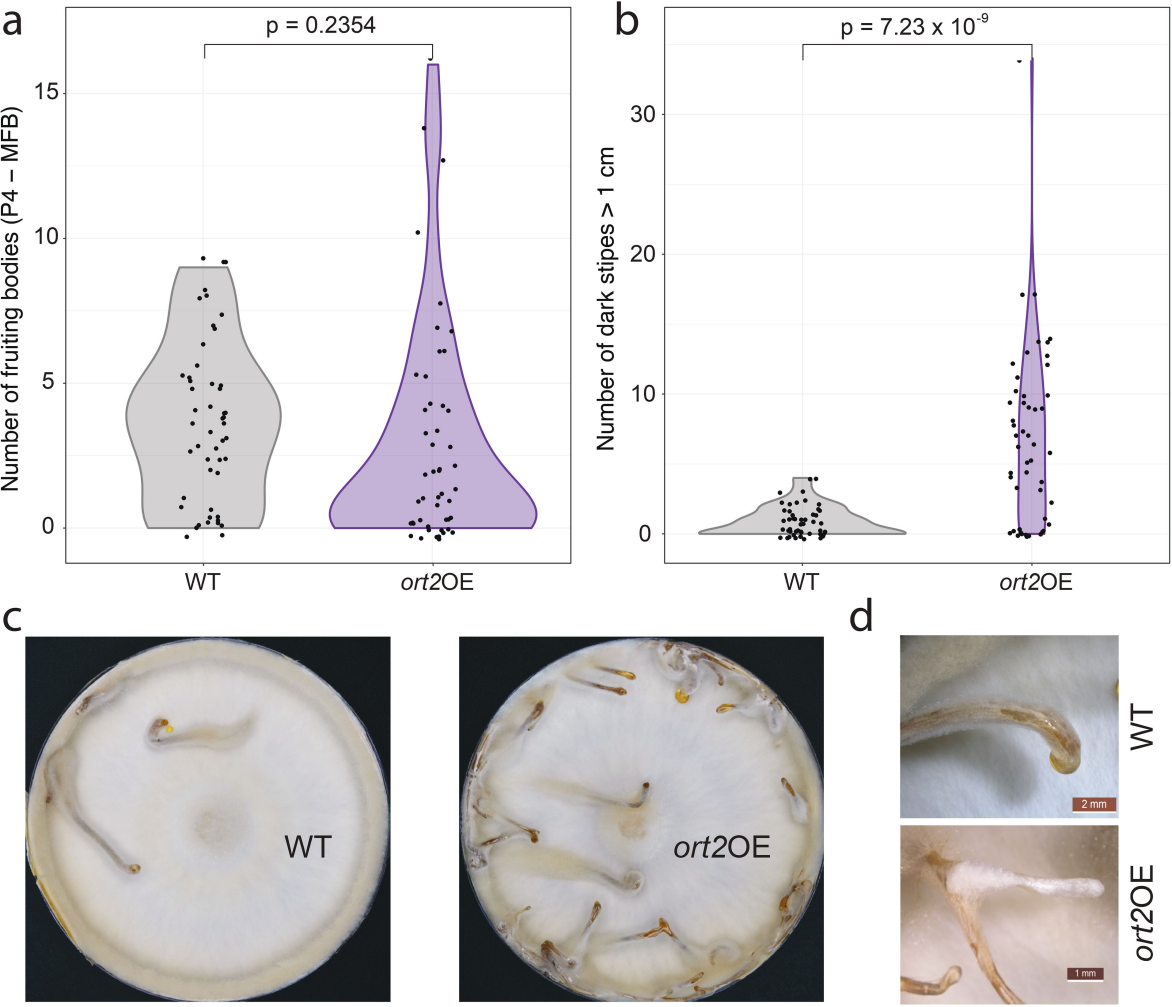
Phenotypic characterization of the *ort2*OE mutants. **(a)** Violin plots illustrating the total number of fruiting bodies (P4 to MFB) on light-grown Petri dish cultures of the wild-type and *ort2*OE strains. **(b)** Violin plots of the number of dark stipes exceeding 1 cm in length, formed by the wild-type and *ort2*OE strains. **(c)** Comparison of WT and *ort2*OE dark-grown cultures. **(d)** An overexpression-related abnormality; the reason we chose *ort2* (two-headed Orthrus) as a name for *C. cinerea* 354109. The branched fruiting bodies were more pronounced in dark stipes (pictured). For other overexpression-related abnormalities, see **Supplementary Figure S3**.

According to our observations, the overexpression of *ort2* did not affect vegetative growth characteristics and oidiation, however, we observed an effect on fruiting. Under alternating light, the *ort2*OE strains are able to complete the fruiting program; from hyphal knots to mature fruiting bodies, all developmental stages were observed, as well as sclerotia and dark stipes. For plates grown under alternating light conditions, we counted structures whose stipes had already started to elongate (P4 to MFB), together. On 50-50 Petri dish cultures, we found that the number of fruiting bodies reaching stage 4 primordium or older was similar between the wild type and overexpression strains (*p* = 0.2354, two-sample t-test) (**Figure 3a**). Mature, deliquescent fruiting bodies were not observed in the wild-type strain during the phenotyping, whereas, on 50 *ort2*OE Petri-dish cultures, altogether 8, and on 4 cultures grown in jars, 6 fruiting bodies were able to complete development by the end of the experiment. In the case of dark-grown colonies, we counted etiolated fruiting bodies taller than 1 cm, as a proxy for the number of dark stipes. The overexpression mutant produced significantly more dark stipes than the wild-type strain (*p* = 7.23 × 10^-9^, two-sample t-test) (**Figures 3b, c**). The average number of these structures per Petri dish was approximately 7.5 times higher in the case of the *ort2*OE mutant than in the wild-type strain. This, combined with the disruptant phenotypes, suggests that *ort2* influences the development of dark stipes.

In addition, the *ort2* overexpression strains exhibit diverse developmental abnormalities, which occur at low frequencies (and therefore cannot be subjected to statistical tests) (**Supplementary Figure S3**). For dark-grown colonies, these phenotypes include the intertwining of etiolated fruiting bodies, forming amorphous structures (**Supplementary Figure S3d**), and the formation of robust prostrate dark stipes. Some of these reached lengths of up to 20 cm, nearly encircling the dish (**Supplementary Figures S3c-d**), a phenomenon that is uncommon in the wild-type strain. Branched fruiting body morphologies, occurring on both dark-grown colonies and those exposed to alternating light, constitute another low-frequency developmental abnormality observed upon *ort2* overexpression (**Figure 3d**; **Supplementary Figure S3a**). We documented fruiting bodies with branched stipes, as well as ones bearing protrusions on their caps (or tips). The branched fruiting bodies and snake-like prostrate dark stipes led us to name *C. cinerea* 354109 *ort2* after Orthrus, the two-headed, snake-tailed dog from classical mythology.

### Mechanistic insights into the function of ort2 are limited

We attempted obtaining mechanistic insights into the role of Ort2 in *C. cinerea*, by (1) functional enrichment analyses of putative targets inferred by a large language model (Karollus et al., 2024) and (2) by specifically addressing whether it is involved in mitochondrial biogenesis, a function that was postulated to be a Saccharomycotina-specific innovation (Jiang et al., 2010).

**Figure 4:**
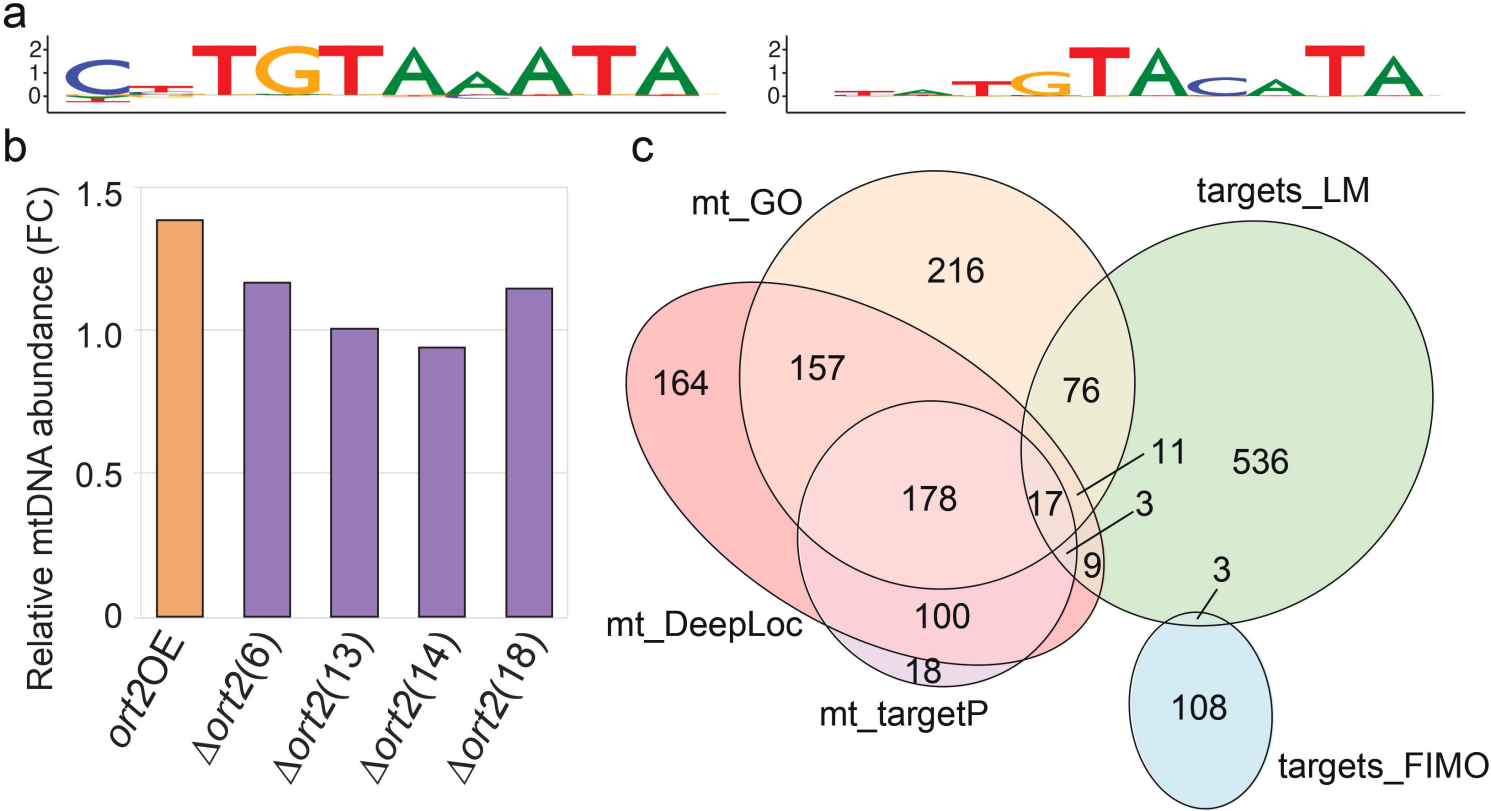
In silico prediction of Ort2 target genes. **(a)** *S. cerevisiae* Puf3p recognition motif (based on (Gerber et al., 2004)) (left) and Ort2 recognition motif predicted by the language model (right). Nucleotide frequencies of the aligned sites were transformed to information content. **(b)** qPCR measurement of mtDNA levels, relative to a nuclear gene, in the Δ*ort2* and *ort2*OE strains, compared to a wild-type control. The relative mtDNA abundance of the wild type corresponds to 1 on the plot. Due to the necessity of averaging during the calculation of FC values, standard deviations of FC values cannot be assessed. See raw data in **Supplementary Table S5**. **(c)** Euler diagram of five *C. cinerea* protein sets: those with mitochondrial GO annotations (mt_GO), proteins with mitochondrial localization predicted by DeepLoc 2.0 (mt_DeepLoc), proteins with mitochondrial localization predicted by TergetP 2.0 (mt_TargetP), Ort2 targets predicted by Karollus et al.’s language model (targets_LM), and Ort2 targets predicted using FIMO (targets_FIMO). See gene sets in **Supplementary Table S6**. Euler plots are model-based, and the model might not include low-cardinality intersections. For the precise overlap analysis, see **Supplementary Figure S4**.

Using a language model (Karollus et al., 2024), reconstruction probability arrays were generated for the *C. cinerea* Okayama 7 genome (Stajich et al., 2010) (300 bps downstream of STOP codons, since Pumilio proteins primarily bind the 3’-UTRs of transcripts (Nishanth and Simon, 2020). Three motifs were identified that significantly matched the *S. cerevisiae* Puf3p motif (using TF-MoDISco and TOMTOM). The most significant motif (**Figure 4a**) was found in 772 C*. cinerea* Okayama 7 transcripts, 655 of which were found in the AmutBmut strainThe information content of the reconstruction probabilities of motif sites was significantly higher than that of sites containing the shuffled motif (*p* = 4.02×10^-166^, Mann-Whitney U test), suggesting the DNA language model recognizes the specific nucleotide arrangement defining the motif, not just general sequence composition. This supports the motif’s robustness and increased confidence in future use. It is worth noting that the central position in the predicted *C. cinerea* motif – where it differs from the *S. cerevisiae* Puf3p motif (A) – can similarly be occupied by a cytosine in the experimentally determined binding site of the *Neurospora crassa* ortholog (Wilinski et al., 2017) (**Figure 4a**), supporting our prediction. We analyzed GO enrichment within the 655 C*. cinerea* AmutBmut target genes. This analysis did not reveal a clear signal, with only five significantly enriched terms (adjusted *p*-value < 0.05, **Supplementary Table S4**). The cause of the lack of signal in this dataset may be manifold, though we note a clear functional enrichment was not found for *N. crassa* either, perhaps meaning that these proteins bind functionally diverse transcripts. Nevertheless, our GO results did not comprise terms related to the mitochondrion, in agreement with previous results on filamentous fungi (Jiang et al., 2010).

To verify this finding, we first compared mtDNA abundance in the Δ*ort2, ort2*OE, and wild-type strains, using quantitative PCR targeting a nuclear (β-tubulin) and a mitochondrially encoded gene (NADH dehydrogenase subunit 1, **Figure 4b**; **Supplementary Table S5**). If Ort2 regulated mitochondrial biogenesis, one would expect overexpression and deletion to have opposite effects on mtDNA abundance. However, no such differences were observed. The measured mtDNA levels in the disruptants were not significantly different from those in the wild type. Although the selected overexpression strain displayed a more pronounced difference, this change occurred in the opposite direction than one would expect based on the role of *S. cerevisiae* Puf3p as a negative regulator of mitochondrial biogenesis (García-Rodríguez et al., 2007).

As an alternative approach, we compared candidate Ort2 targets inferred by *in silico* methods to mitochondrial proteins of *C. cinerea* (1) Gene Ontology (GO) annotations, (2) subcellular protein localization predicted by DeepLoc 2.0, and (3) by TargetP 2.0. The three mitochondrial gene sets considerably overlap (**Figure 4c**), validating these predictions. Mitochondrial protein lists are provided in **Supplementary Table S6**. However, when these gene sets were compared with 655 putative Ort2 targets predicted by the language model, and with 116 C*. cinerea* mRNA targets predicted with FIMO (using the *S. cerevisiae* Puf3p recognition motif as query), we obtained small overlaps (**Figure 4c**; **Supplementary Table S6**). Only one comparison, between the set of genes with mitochondrial GO annotation and the list of Ort2 targets predicted by the language model, revealed a significant overlap: 14.77% of the LM hits are in the GO set (*p* < 10^-5^, Fisher’s, exact test).

Prediction of the subcellular localization of *ort2* using in silico tools suggested that *ort2* is cytoplasmic and nuclear. DeepLoc 2.0 yielded scores of 0.7543 and 0.5299 for cytoplasmic and nuclear localization, respectively, with much lower (>0.09) scores for other localizations. WolfPsort ranked a nuclear localization first (score: 13), followed by cytoplasmic (6), mitochondrial (4), and peroxisomal (3). We hypothesize that *ort2* may shuttle between the cytoplasm and the nucleus, possibly depending on posttranslational modifications. This is consistent with the behavior of other Pumilio family proteins (Gerber et al., 2004). Overall, we interpret these results as an absence of evidence for a mitochondrial function of Ort2 is in line with the data of Jiang et al. (2010) on filamentous Ascomycota. It remains to be tested whether the predicted mRNA targets are biologically significant and how these contribute to fruiting and dark stipe development remains unclear.

## Discussion

In this study, we examined the Pumilio family of RNA-binding proteins in mushroom-forming fungi and addressed the role of a conserved member of the family, *ort2*, selected based on its expression patterns during mushroom formation. Our experiments show that *ort2* influences both fruiting in the light and dark stipe development, with a stronger effect on the latter. Since no effect on vegetative morphology was observed, the role of *ort2* is likely to be specific to sexual development.

Phylogenetic analyses indicated that the Pumilio family displays a remarkable internal structure, with clearly discernible clades, which may be interpreted as subfamilies. Each of these clades showed a high level of sequence conservation, and orthology relationships were conserved with a single copy from most species. This is in contrast with the much more dynamic evolution of the family at the scale of all eukaryotes (Najdrová et al., 2020), suggesting that within fungi, there is a higher level of Pumilio conservation. On the other hand, our data indicate that the conservation of function might not follow the conservation of sequence and orthology. Despite being a close ortholog of *S. cerevisiae PUF3*, our results do not support *ort2* having a similar, mitochondrion-related function, which may be due to fast evolutionary turnover of function, despite a high level of sequence conservation, as has been described for RNA-regulatory networks (Wilinski et al., 2017).

The deletion of *ort2* resulted in deficient dark stipe development, while its overexpression significantly increased their number. At this point, we lack a mechanistic explanation for the described qualitative and quantitative phenotypes. Dark stipe formation is a complex developmental program that remains largely unexplored. Hitherto, molecular insights into dark stipes have been limited exclusively to photoreception (Subba, 2022; Chaisaena, 2009). Given that our *ort2* mutants are not ‘blind’, and dark stipe formation is more profoundly affected than fruiting body formation in light, we suggest that it acts independently of light-regulated pathways and that further investigation of *ort2* might illuminate other aspects of dark stipe formation. Since dark stipes arise as a result of the growth of the basal plectenchyma (nodulus) (Terashima et al., 2005), it is tempting to hypothesize that *ort2* contributes to nodulus functioning; nonetheless, further investigation is needed. We acknowledge the lack of a supported mechanistic explanation behind the described phenotypes and propose conducting transcriptomic analyses of *ort2* mutants to gain more knowledge about the regulatory function of Ort2.

Our findings might have various practical applications. The overexpression of *ort2* resulted in the higher abundance of dark stipes. There are popular edible species whose etiolated fruiting bodies are consumed – one such species, *F. velutipes* is among those with the greatest economic importance (Rugolo et al., 2016). Given our results, overexpression of the *F. velutipes ort2* ortholog may be worth exploring from a commercial perspective. If the function is conserved, these findings may be applicable in mushroom cultivation. Furthermore, in our experiments fruiting bodies of the *ort2* overexpression strain more frequently reached maturity than the parent strain. The unpredictability and varying success of fructification of different fungal species, even in a controlled environment (Prayook et al., 2013), pose a major challenge for the developmental biology of fruiting bodies. In our experience, even fruiting bodies of the *C. cinerea* AmutBmut strain frequently arrest their development in a laboratory environment. We suggest that for future fruiting body research, especially for studies focusing on late stages or the entire developmental process, the *ort2*OE strain might be a more suitable model system than the AmutBmut parent strain.

Ort2 is orthologous with Pum1 of *Cr. neoformans*, and, though RNA regulatory networks rewire quickly during evolution (Wilinski et al., 2017), a functional comparison between the two genes is of interest. Pum1 was postulated to be a pleiotropic regulator that positively influences basidium maturation and is involved in maintaining filamentous growth in *Cr. neoformans* (Liu et al., 2018; Kaur and Panepinto, 2016). It is notable that, at the highest level, both *ort2* and *PUM1* influence sexual development. The vastly different sexual development of these two species - *Cr. neoformans* produces simple basidial lawns (Kwon-Chung, 1976), whereas *C. cinerea* basidia are enclosed in complex fruiting bodies - currently precludes a more detailed comparison, however, it is possible that these genes regulate similar pathways or mechanisms. Further studies and comparisons of target gene sets in *C. cinerea* with those of *Cr. neoformans* are required to address these questions.

Ort2 is the first RNA-binding protein with a documented role in fruiting body development. Given that Pumilio proteins have been shown to possess developmental significance in animals and plants (Nishanth and Simon, 2020), further characterization of RNA regulatory networks in fungi may illuminate novel aspects of complex multicellularity in fungi. We anticipate that the characterization of *ort2*, reported here, will be useful in charting this new realm of fungal developmental regulation. Experimental identification of Ort2 targets might provide mechanistic explanations and an interesting future research direction.

## Data Availability

The original contributions presented in the study are included in the article/**Supplementary Material**. Further inquiries can be directed to the corresponding author.

## Supplementary material

The Supplementary Material for this article can be found online at: https://www.frontiersin.org/articles/10.3389/ffunb.2025.1633301/full#supplementary-material

Supplementary Figure S1 Rooted gene tree of fungal Pumilio proteins, with bootstrap values. The phylogenetic analysis comprises protein sequences from 17 fungal species. All terminal nodes are labeled. The five clades are colored differently.

Supplementary Figure S2 Plasmids used for genetic modification of *C. cinerea*. **a**. We used plasmid p.Δ*ort2* to generate disruptants. Abbreviations: CcPAB1 cassette - selection marker amplified from the pMA412 vector (Stanley et al., 2014), UHA and DHA - upstream and downstream homologous arms, and pUC19 backbone. **b**. We used plasmid p.*ort2*OE to generate overexpression mutants. Parts of the construct: CcPAB1 cassette, *hsp3* (CopciAB_423239) promoter, *ort2* without its own promoter, and pUC19 backbone.

Supplementary Figure S3 Low-frequency developmental abnormalities of the *ort2* overexpression mutants. **a**. Branched fruiting bodies. Top row: fruiting bodies formed under alternating light/dark conditions; bottom row: etiolated fruiting bodies. Two photographs on the left represent the wild-type strain, the other four are *ort2*OE fruiting bodies. **b-c**. Prostrate etiolated fruiting bodies formed by the *ort2*OE strains. Blue arrows follow a dark stipe through the edge of the dish. **d**. *ort2*OE dark stipes usually intertwine and form amorphous structures. Note that at the base of the structure in the picture, individual dark stipes could not be distinguished.

Supplementary Figure S4 UpSet plot for the overlap analysis of five *C. cinerea* AmutBmut gene sets: genes with mitochondrial GO annotations (mt_GO), proteins with mitochondrial localization predicted by DeepLoc 2.0 (mt_DeepLoc), proteins with mitochondrial localization predicted by TergetP 2.0 (mt_TargetP), Ort2 targets predicted by Karollus et al.’s language model (targets_LM), and Ort2 targets predicted using FIMO (targets_FIMO).

Supplementary Table S1 Sequences of crRNAs used for the disruption through Cas9 cleavage and primers used for plasmid construction, transformant screening, and qPCR.

Supplementary Table S2 Raw qPCR data for the examination of *ort2* expression in Δ*ort2* disruptants.

Supplementary Table S3 Raw qPCR data and fold-changes in *ort2* expression in *ort2*OE strains relative to the parent strain.

Supplementary Table S4 Gene Ontology terms significantly (*p* < 0.05) enriched among putative Ort2 targets belonging to the most significant recognition motif predicted by the species-aware language model.

Supplementary Table S5 Raw data and calculation of relative mtDNA abundance in *ort2* mutants.

Supplementary Table S6 The five gene sets used for functional studies: genes with mitochondrial GO annotations (mt_GO), proteins with mitochondrial localization predicted by DeepLoc 2.0 (mt_DeepLoc), proteins with mitochondrial localization predicted by TergetP 2.0 (mt_TargetP), Ort2 targets predicted by Karollus et al.’s language model (targets_LM), and Ort2 targets predicted using FIMO (targets_FIMO). targets_LM and mt_GO are provided with both Okayama and AmutBmut IDs, too. In both cases, the datasets containing Okayama identifiers are the original, and they were converted to AmutBmut, to align with the other three gene sets, resulting in the loss of some genes. For the comparison between targets_LM and mt_GO, Fisher’s exact test was performed using the lists containing Okayama IDs; for comparisons across all five datasets, the *C. cinerea* AmutBmut lists were used.
