# Supplementary figures and images for: Orthrus: a Pumilio-family gene involved in fruiting body and dark stipe development in Coprinopsis cinerea
Source: Front Fungal Biol. 2025 Jul 30;6:1633301. doi: 10.3389/ffunb.2025.1633301 (PMC12344735; doi:10.3389/ffunb.2025.1633301)

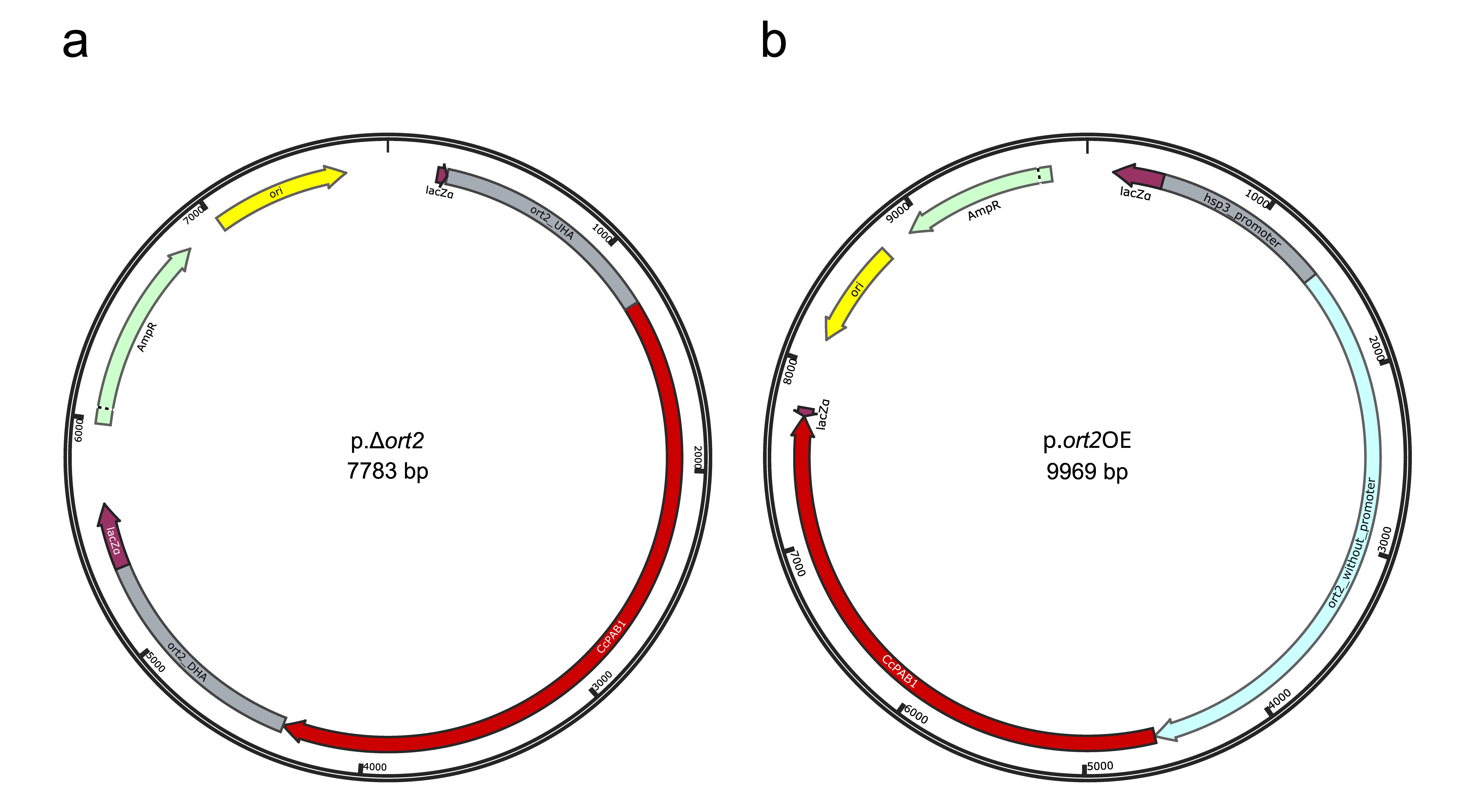

Supplement: Supplementary Figure S2 — Plasmids used for genetic modification of C. cinerea. a. We used plasmid p.Δort2 to generate disruptants. Abbreviations: CcPAB1 cassette - selection marker amplified from the pMA412 vector (Stanley et al., 2014), UHA and DHA - upstream and downstream homologous arms, and pUC19 backbone. b. We used plasmid p.ort2OE to generate overexpression mutants. Parts of the construct: CcPAB1 cassette, hsp3 (CopciAB_423239) promoter, ort2 without its own promoter, and pUC19 backbone. [file Image2.jpg]

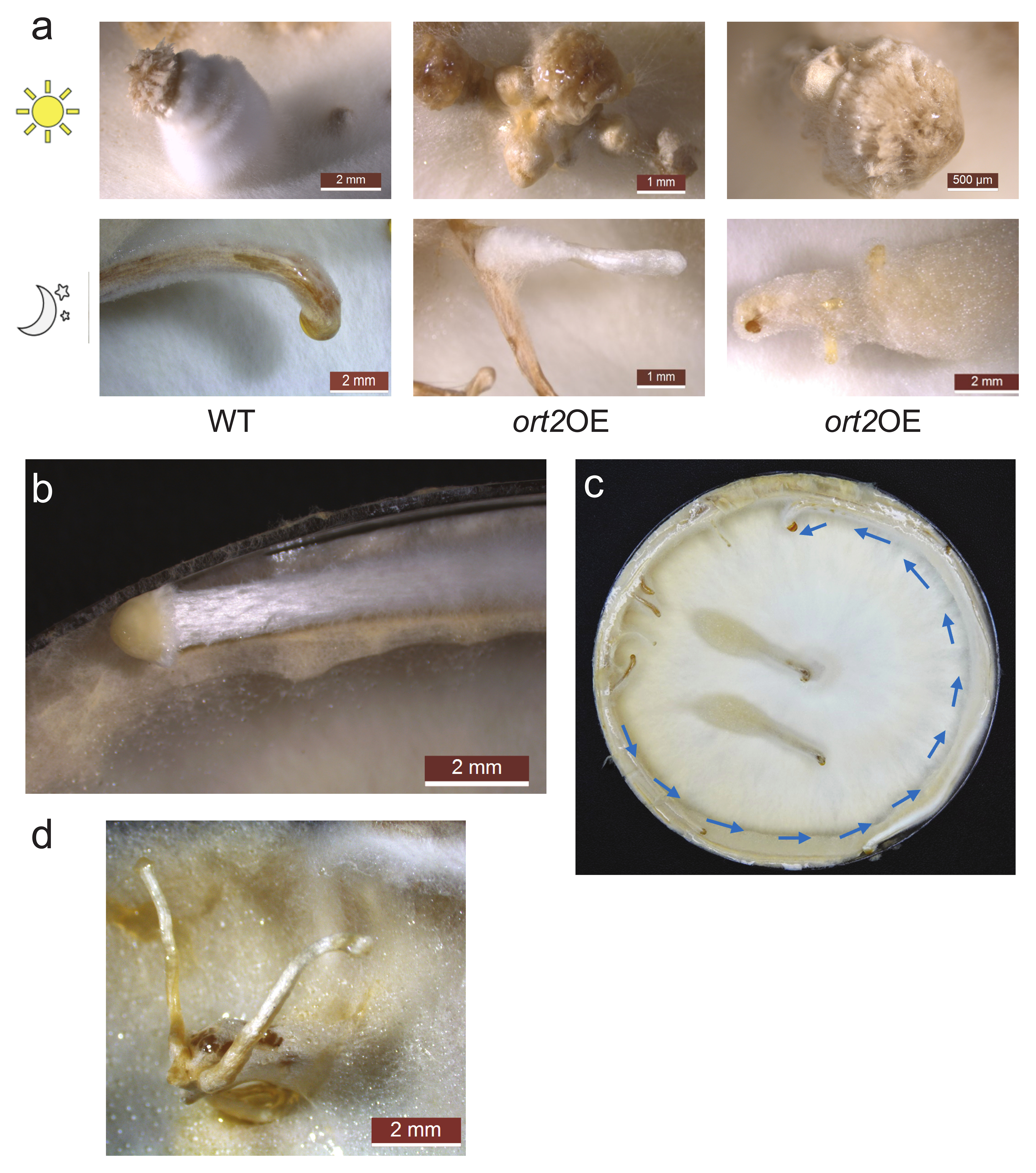

Supplement: Supplementary Figure S3 — Low-frequency developmental abnormalities of the ort2 overexpression mutants. a. Branched fruiting bodies. Top row: fruiting bodies formed under alternating light/dark conditions; bottom row: etiolated fruiting bodies. Two photographs on the left represent the wild-type strain, the other four are ort2OE fruiting bodies. b-c. Prostrate etiolated fruiting bodies formed by the ort2OE strains. Blue arrows follow a dark stipe through the edge of the dish. d. ort2OE dark stipes usually intertwine and form amorphous structures. Note that at the base of the structure in the picture, individual dark stipes could not be distinguished. [file Image3.jpg]

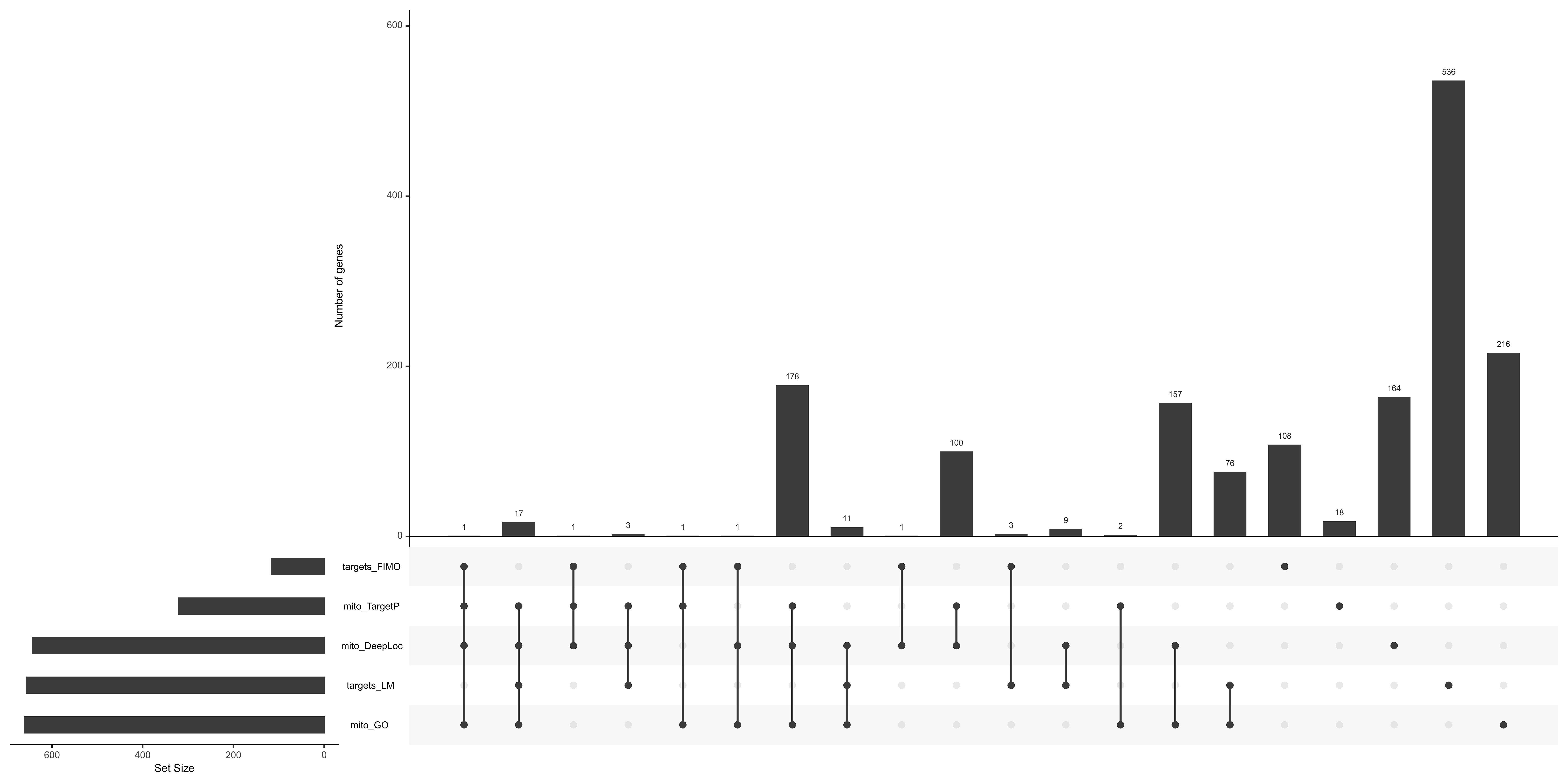

Supplement: Supplementary Figure S4 — UpSet plot for the overlap analysis of five C. cinerea AmutBmut gene sets: genes with mitochondrial GO annotations (mt_GO), proteins with mitochondrial localization predicted by DeepLoc 2.0 (mt_DeepLoc), proteins with mitochondrial localization predicted by TergetP 2.0 (mt_TargetP), Ort2 targets predicted by Karollus et al.’s language model (targets_LM), and Ort2 targets predicted using FIMO (targets_FIMO). [file Image4.jpg]
